# Rhythm Generation and Rhythm Perception in Insects: The Evolution of Synchronous Choruses

**DOI:** 10.3389/fnins.2016.00223

**Published:** 2016-05-31

**Authors:** Manfred Hartbauer, Heiner Römer

**Affiliations:** Behavioural Ecology and Neurobiology, Institute of Zoology, University of GrazGraz, Austria

**Keywords:** insect choruses, chorus synchrony, female choice, rhythm generation, pattern recognition, cooperation

## Abstract

Insect sounds dominate the acoustic environment in many natural habitats such as rainforests or meadows on a warm summer day. Among acoustic insects, usually males are the calling sex; they generate signals that transmit information about the species-identity, sex, location, or even sender quality to conspecific receivers. Males of some insect species generate signals at distinct time intervals, and other males adjust their own rhythm relative to that of their conspecific neighbors, which leads to fascinating acoustic group displays. Although signal timing in a chorus can have important consequences for the calling energetics, reproductive success and predation risk of individuals, still little is known about the selective forces that favor the evolution of insect choruses. Here, we review recent advances in our understanding of the neuronal network responsible for acoustic pattern generation of a signaler, and pattern recognition in receivers. We also describe different proximate mechanisms that facilitate the synchronous generation of signals in a chorus and provide examples of suggested hypotheses to explain the evolution of chorus synchrony in insects. Some hypotheses are related to sexual selection and inter-male cooperation or competition, whereas others refer to the selection pressure exerted by natural predators. In this article, we summarize the results of studies that address chorus synchrony in the tropical katydid *Mecopoda elongata*, where some males persistently signal as followers although this reduces their mating success.

## Acoustic communication in insects

Grasshoppers, crickets, and katydids usually produce sound by stridulation, that is using a striated file-like body structure and associated structures that vibrate when they are rubbed across a sclerotized plectrum (peg). While crickets and katydids rub their forewings against each other, grasshoppers move their hind legs across a peg located at the base of their wings. The sound signals generated can be as short as 0.5 ms (i.e., the female acoustic reply in Phaneropterine species) or can last for many minutes or even longer (e.g., the calling songs of trilling katydids). Acoustic signals can also be classified according to the responses they evoke from conspecific receivers: signals that are generated in aggressive interactions with conspecific rivals are termed aggressive songs, whereas calling songs are used to attract mates (Heller, 1988). When within close range to females, males often generate courtship songs with reduced amplitudes, different temporal patterns, and carrier frequencies. In most species, only males generate acoustic signals, and the mute females approach the singing males (phonotaxis). In duetting species, females reply to signals produced by distant males by emitting a short acoustic signal, which then elicits male phonotaxis (Heller and von Helversen, 1986; Zimmermann et al., 1989). A general feature of acoustic signals in insects is their high degree of stereotypy and redundancy. Since acoustic signals serve as effective premating isolation barriers, they are highly diverse among species. The temporal signal pattern is particularly essential for species recognition among grasshoppers (von Helversen and von Helversen, 1975, 1998), katydids (e.g., Morris et al., 1975; Keuper and Kühne, 1983), and crickets (e.g., Walker, 1957, 1969; Popov and Shuvalov, 1977; Mhatre et al., 2011; Schmidt and Römer, 2011; Schmidt and Balakrishnan, 2015). The carrier frequencies can range from 1 to 2 kHz far into the ultrasonics, and signals can be broadband (as in many katydids) or fall within a narrow frequency band (most crickets). The selective advantage of using either broadband or narrow-band acoustic signals for sound transmission and perception in a noisy environment has been previously described (Rheinlaender and Römer, 1980; Schmidt and Römer, 2011; Schmidt et al., 2011, 2013; Schmidt and Balakrishnan, 2015).

After successfully detecting signals, receivers evaluate the temporal signal pattern to obtain information about the species identity of the signaler. When signal period is rather variable or males advertise themselves by producing long-lasting trills, the period of *syllables* (for definition, see Table 1) usually contains information about the species identity (e.g., Walker, 1957; Popov and Shuvalov, 1977; Doherty and Callos, 1991; Simmons, 1991; Cade and Cade, 1992). However, when males generate a group of syllables (termed *chirps*) at fixed time intervals, the signal period could be a cue that indicates species identity (e.g., Walker, 1969). With reference to the current topic of timing in music and speech, the latter is particularly important. The intrinsic signal period of males shows little variability in some acoustic insect species, and males listen and respond to the signals of conspecific neighbors. As a result, the signal timing of chorus members strongly deviates from random, whereby synchrony and signal alternation are extreme forms of temporal patterns that emerge from acoustic interactions. Since signal timing in a group can have important consequences for calling energetics, mate choice, and predation, researchers have been asking questions about the evolution of chorusing for decades. Before going into detail about the various causes and consequences of synchronous insect choruses, we will provide a brief review of recent advances in our understanding of the neuronal basis of signal pattern generation and rhythm perception in insects, both of which are basic requirements for acoustic communication.

**Table 1:** **Definition of bioacoustic terms**.

**Term**	**Temporal pattern**	**Duration**
Syllable	Unitary element of chirps	5–30 ms
Chirp	Consists of several syllables	50–800 ms
Trill	Consists of a train of syllables	Minutes to hours

### Rhythm-generating neural circuits

The temporal patterns of acoustic signals are generated by rhythm-generating networks of the central nervous system. Acoustic insects are valuable model organisms for the study of these networks because the rhythm of their songs is rather simple and their nervous system is rather primitive as compared to vertebrates or mammals. Another advantage is that neurons can be identified on the basis of their response properties and unique anatomy. This allows comparisons of the function of identified homologous neurons that are part of pattern-generating networks across species to be made, which provides important insights into the evolution of both temporal signal patterns and song diversification.

In order to attract females from a distance, males of the Mediterranean field cricket *Gryllus bimaculatus* emit calling songs that are characterized by aperiodic chirps consisting of about 4–5 syllables. Recently, the network involved in pattern generation was identified in this species. Schöneich and Hedwig (2011) located its position in the CNS by systematically dissecting the connection between abdominal ganglia (for a similar method, see Hennig and Otte, 1996). After transecting the connectives between the third thoracic ganglion (metathoracic ganglion complex) and the first abdominal ganglion, singing behavior was immediately and permanently terminated. Later, four neurons in these ganglia that showed rhythmic activity in phase with the syllable pattern were identified (Schöneich and Hedwig, 2012). Interestingly, a similar, characteristic neuroanatomy of the song pattern generator was found in the metathoracic-abdominal ganglion complex in grasshoppers, where songs are produced through rhythmic movements of hind legs (Gramoll and Elsner, 1987; Hedwig, 1992; Schütze and Elsner, 2001). Even more surprising, the neuronal circuit for courtship song production in drosophila (Clyne and Miesenböck, 2008; von Philipsborn et al., 2011) and rhythmic sound production via tymbals in arctiid moths (Dawson and Fullard, 1995) was also located in thoracic-abdominal ganglia. This suggests a common evolutionary origin for early thoracic-abdominal motor control networks, which may have been linked to ventilation (cf. Robertson et al., 1982; Dumont and Robertson, 1986). By gathering knowledge about the location and function of interneurons that constitute part of the central pattern generator, a framework for further comparative studies can be constructed. In such an attempt it would be worthwhile to investigate the neuronal basis that is responsible for rhythm adjustment in chorusing insects (see below).

### Rhythm perception and associated neuronal correlates

Mate choice experiments performed with various field cricket and katydid species have revealed that the signal traits evaluated by receivers for species recognition are as diverse as the signals (e.g., Heller and von Helversen, 1986; Shaw et al., 1990; Simmons, 1991; Hennig and Weber, 1997; Hennig, 2003, 2009; Poulet and Hedwig, 2005; Greenfield and Schul, 2008; Hartbauer et al., 2014; Hennig et al., 2014). It has been generally accepted that temporal pattern recognition is both hardwired and genetically-determined as compared to olfaction and visual orientation, where learning also plays an important role (Bazhenov et al., 2005; Papaj and Lewis, 2012). To understand the principal mechanisms of species recognition and mate choice in insects, it is necessary to unravel the response properties both of auditory neurons that convey information about acoustic signals to the brain, and the filter network in the brain itself. The expectation in this research was to find a neuronal network and describe synaptic mechanisms that result in selective responses to the conspecific temporal song pattern, which matches the selectivity of these patterns in behavior. Two model organisms were used for this approach: the grasshopper *Chorthippus biguttulus* and the field cricket *G. bimaculatus*.

Male *Ch. biguttulus* grasshoppers generate temporally-structured signals via stridulation and females respond to the temporal pattern of syllable-pause combinations of attractive songs by emitting a short acoustic reply (von Helversen and von Helversen, 1998; Meckenhäuser et al., 2013). Females in this species prefer short pauses and a strong onset accentuation of song elements (von Helversen, 1972; Balakrishnan et al., 2001). Stumpner et al. (1991) studied the response of several neurons to conspecific song models and showed that, of various local neurons in the thorax, one neuron (BSN1) responded to varying syllable-pause combinations in a way that matched behavior. Two other thoracic neurons (SN6, AN4) responded to gaps in the verse of conspecific song models in a highly reliable manner (Stumpner and Ronacher, 1994). By selectively heating individual body segments, the brain was identified as the location where pattern recognition takes place, whereas the oscillator for song production was localized in the thoracic ganglia (Bauer and von Helversen, 1987; Gramoll and Elsner, 1987; Hedwig, 1992; Schütze and Elsner, 2001; Schöneich and Hedwig, 2012). The brain neurons involved in pattern recognition still need to be characterized in this species.

As already mentioned above, male *G. bimaculatus* attract distant females by producing calling songs that are made up of aperiodic chirps, each consisting of about four syllables. As in many other cricket species, the syllable period represents a crucial parameter for species recognition. Behavioral experiments revealed that song pattern recognition in *G. bimaculatus* relies on two computations with respect to time (Grobe et al., 2012). Using a modern modeling approach, Hennig et al. (2014) were able to simulate the response of females that listened to various calling song models with different temporal patterns by using a short integration time window that operated as a filter for the pulse rate and a longer integration time window that allowed the evaluation of song energy over time.

**Figure 1:**
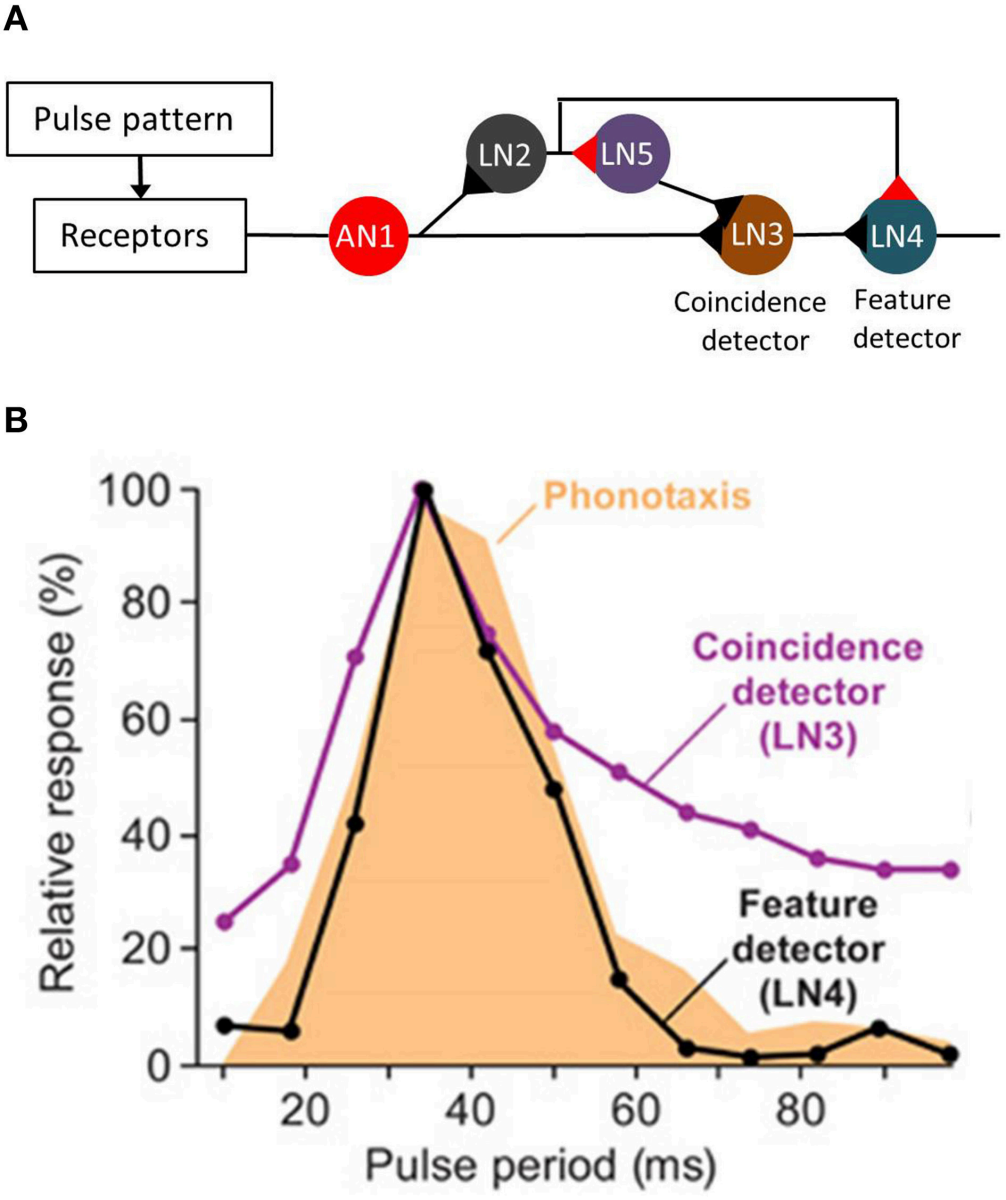
**Pulse pattern recognition in ***G. bimaculatus***. (A)** Schematic drawing of the feature detector involved in the recognition of pulse rates. Black triangles represent the excitatory synapses and red triangles, the inhibitory synapses. LN2 and LN5 caused a delay of the nervous response in LN3 relative to the excitation mediated via AN1. **(B)** The pulse period tuning of the feature detector LN4 matched female preference. Modified from Schöneich et al. (2015).

Recently, the neuronal network that enables pulse rate recognition in the brain of *G. bimaculatus* has been identified. It turned out that this complex task depends on the detection of the coincidence of successive pulses in a delay line network (Schöneich et al., 2015). Subsequent sound pulses are encoded in the bursting activity of a neuron that receives sensory input at the thorax and ascends to the brain (AN1). In the brain, the sensory information of this neuron is split into two parallel pathways, one involving two other neurons (LN2 and LN5). The processing of sensory information in these neurons leads to a moderate delay and, thus, to the coincidence of the bursting response of AN1 and LN5 in the postsynaptic neuron LN3 when pulses are separated by a syllable interval of more than 20 ms (see Figure 1A). While LN3 operates as coincidence detector, LN4 represents a feature detector that exhibits temporal band pass characteristics that are highly similar to those of the pulse period tuning of female phonotaxis (Figure 1B; Kostarakos and Hedwig, 2012). This feature-detection mechanism enables recognition of the species-specific temporal song pattern in this field cricket, and is a principal mechanism that evaluates the pulse period of calling songs.

## Insect choruses

In some insect species, males congregate in groups where they form acoustic *leks* (also referred to as “spree” in the temporal domain; Walker, 1983; Kirkpatrick and Ryan, 1991; Höglund and Alatalo, 1995). These male aggregations offer females the opportunity to compare the calling songs of several males simultaneously, which is principally different from sequentially comparing potential mating partners (Kokko, 1997). An analysis of signal timing in males within these aggregations revealed various forms of collective broadcasting where signal timing was non-random. Greenfield (1994a) reviewed various mechanisms for a joint display of signals in groups. These included: (1) Changing light conditions trigger the simultaneous activity of senders in dusk and dawn choruses. (2) Unison bout singing, triggered by males who initiate calling and are then joined by most other signalers. Participants in such choruses usually maintain a high signal rate for several minutes, after which the calling effort gradually decreases to zero. Then, the cycle is repeated after variable intervals of silence. (3) Periodic signal production can be controlled through a central pattern generator that leads to high precision of signal timing, if individuals in a group slowly adapt their signal period to the rhythm of others who exhibit similar, intrinsic, “free-running” signal periods (as in some synchronizing firefly species). (4) In some chorusing species, males are able to maintain a constant phase relationship between their signals and those of other males by responding with a phase shift to the signal produced by a neighbor. Depending on certain properties of signal oscillators and the number of participants, signals are either broadcast in collective synchrony or in a kind of alternation.

When singing within the hearing range of one other, males of the same species often time their signals strictly and temporally. Depending on chorus size and inter-male distance, males either alternate (e.g., Jones, 1963; Latimer, 1981; Meixner and Shaw, 1986; Tauber et al., 2001) or synchronize their periodic signals (Walker, 1969; Shaw et al., 1990; Sismondo, 1990; Greenfield and Roizen, 1993; Nityananda and Balakrishnan, 2007; Greenfield and Schul, 2008; Schul et al., 2014). Synchrony is often found in species that emit signals relatively rapidly (with a period of < 1 s), whereas alternation normally involves slower signal rhythms (a period of >1 s) (Greenfield, 1994b). In principle, alternation in periodic signals is restricted to only two signalers, whereas the number of individuals engaged in synchronous signaling is theoretically unlimited. Depending on the properties of song oscillators, synchrony can either lead to a significant overlap in signals or temporally-fixed delays of signals produced by different males. At close range, synchrony can be rather precise, so that even the syllables within the chirps are synchronized with those of neighboring males: when singing in close proximity, males of the chorusing species *Amblycorypha parvipennis* tend to synchronize the syllable pattern of their signals (Shaw et al., 1990). Synchronous signal displays are not restricted to the acoustic world, but can also be found in other modalities. Aggregating firefly species collectively broadcast visual displays in almost perfect synchrony, which results in fascinating group displays (Buck and Buck, 1968; Otte and Smiley, 1977; Buck et al., 1981). Furthermore, the vibratory communication signals of wolf spiders (Kotiaho et al., 2004) and the visual communication system of fiddler crabs (Backwell et al., 1998) are characterized by their high degree of synchrony.

Is there a common proximate mechanism that is responsible for synchronous signaling in these different systems? The oscillator properties that lead to synchronous signal displays were first described for fireflies, where a “phase delay model” was suggested to explain flash synchrony in these organisms (Hanson, 1978; Buck et al., 1981). Greenfield (1994b; see also Greenfield et al., 1997) modified this model, hypothesizing the existence of an inhibitory resetting mechanism of signal oscillators to explain the diversity of alternating and synchronous choruses observed among members of the different species. In this model, in the absence of a stimulus, the oscillator level constantly rises to a point where the production of a signal is triggered with a minor delay (effector delay). One important characteristic of this model is that the oscillator level is reset for the duration of the stimulus, which leads to a phase delay. However, the neuronal basis of this model has not yet been described.

While inhibitory resetting can lead to the rapid synchronization of signals in a chorus (e.g., *Mecopoda elongata*: Sismondo, 1990; Hartbauer et al., 2005), the degree of synchrony is much higher when the signalers mutually adjust their intrinsic signal rates. Mutual rhythm adjustment has been observed to lead to the attainment of almost perfect flash synchrony in firefly individuals (Ermentrout, 1991). Furthermore, a combination of inhibitory resetting and period adjustment is responsible for the high degree of signal overlap among chorusing katydids (Walker, 1969; Nityananda and Balakrishnan, 2007; Murphy et al., 2016). In the same way, perfect synchrony of humans has been attributed to both “phase correction” and “period adjustment” mechanisms (e.g., Semjen et al., 1998; Repp, 2001, 2005; see also Merker et al., 2009).

### Evolution of chorus synchrony

How synchrony among different individuals could evolve in the absence of a central controlling instance within the group (i.e., an individual that would play a role similar to that of a conductor in an orchestra) is puzzling. Mechanisms that would ultimately favor the evolution of chorus synchrony are thought to be diverse and may have evolved in response to selective forces either driven by other chorus members, through female choice (see Section Female Choice and the Evolution of Chorus Synchrony) or natural predators (see Section Cooperation, Competition, and a Trade-Off between Natural and Sexual Selection). Males that advertise themselves in a chorus may gain one or more of the following mutual (group) benefits by timing signals (reviewed in Greenfield, 1994b): (1) Synchrony preserves a species-specific rhythm or a distinct call envelope that is offset by silent gaps (Walker, 1969; Greenfield and Schul, 2008). (2) In contrast, alternation ensures that females can detect, and discriminate critical signal features during mate choice. (3) Synchrony maximizes the peak signal amplitude of group displays, which is an emergent property also known as the “beacon effect” in the firefly literature (Buck and Buck, 1966, 1978). This property increases the conspicuousness of signals in a group of males as compared to that of a lone singer if females evaluate the peak signal amplitude rather than average signals over a longer period of time. This hypothesis states that males in a group can attract females from a greater distance by timing their signals to achieve nearly perfect synchrony. As a consequence, individuals in a chorus potentially increase their fitness as compared to isolated singing individuals. However, empirical evidence for the existence of a “beacon effect” in acoustic insects is rare and has been restricted to evidence from computer-model simulations of chorus synchrony evolution in an Indian *Mecopoda* species (Nityananda and Balakrishnan, 2009). A strong increase in the amplitude of synchronous acoustic signals was described in *M. elongata* (Hartbauer et al., 2014). For a description of other suspected “beacon effects” in bullfrog choruses see Bates et al. (2010) and in the vibratory communication of a treehopper, see Cocroft (1999). Whereas the hypotheses described above are based on sexual selection, the timing of communal displays may also be shaped by natural selection. For example, predators eavesdropping on the calling songs of signalers may have difficulty localizing an isolated signaler in a group of synchronously-signaling individuals due to their cognitive limitations (Otte, 1977; Tuttle and Ryan, 1982). In this way, males may benefit from a reduced per-capita rate of predation by signaling in groups (Lack, 1968; Wiley, 1991; Alem et al., 2011; Brunel-Pons et al., 2011).

**Figure 2:**
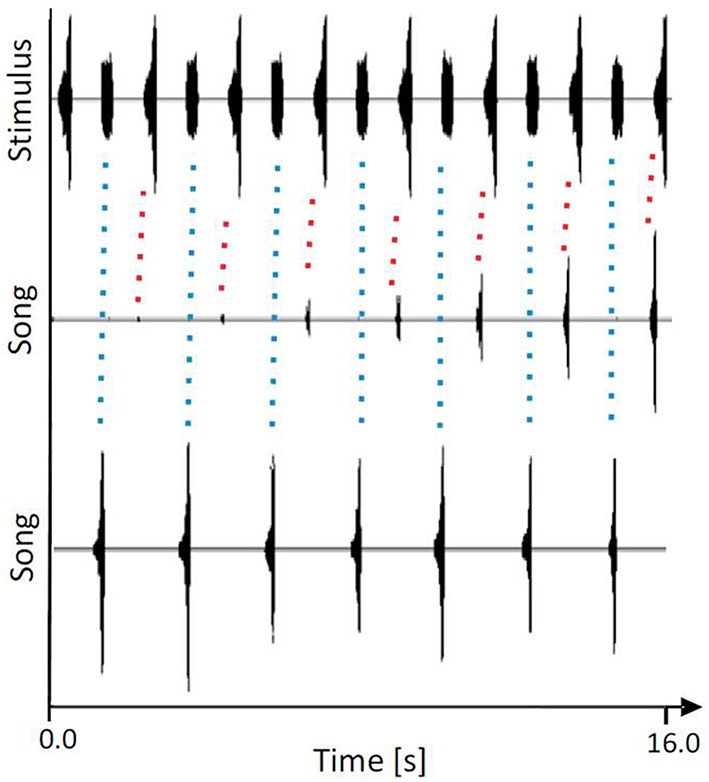
**Representative example of a ***M. elongata*** male being offered the choice to produce chirps either in synchrony with periodic conspecific chirps (higher peak amplitude in the upper trace) or white noise pulses (lower peak amplitude), presented in alternation**. Note that both signals exhibited the same acoustic energy. Middle panel: song initiation. Lower panel: stable entrainment. Note the phase-locking to the chirp that was observed at the onset of the song (indicated by red-dotted lines), but was thereafter observed in synchrony with the artificial pulse (indicated by blue-dotted lines). Modified from Hartbauer et al. (2012b).

The “rhythm conservation” hypothesis and the “beacon effect” hypothesis are not mutually exclusive in that they both explain the evolution of chorus synchrony in male assemblages as a result of inter-male cooperation. The first hypothesis assumes a low amount of variability in the signal period on a species level and suggests that this signal parameter includes important information about species identity, whereas the temporal pattern of syllables that make up chirps is considered to be less relevant. This assumption was recently tested using the katydid species *M. elongata* from Malaysia, males of which synchronize their periodic signals with a period of about 2 s in small choruses (Sismondo, 1990). Calling songs in this species consist of regular chirps that are made up of about 10 syllables increasing in amplitude. When individual males were allowed to synchronize with periodic white noise signals that lacked any fine-temporal pattern, about 80% of males succeeded as long as the signal period was limited to about 2 s (Hartbauer et al., 2012a). Similarly, males synchronized with a periodic stimulus that consisted of only three syllables. In another experiment, individual males were allowed to either signal in synchrony with a conspecific signal or an artificial, unstructured white noise signal, both of which were presented at 2 s intervals and of equal intensity. Interestingly, 65% of the males generated chirps in synchrony with the conspecific signal, whereas only 35% synchronized with the unstructured signal (see example in Figure 2). However, after introducing a phase transition by delaying the stimulus for 1 s, only 56% of chirps were produced in synchrony with the conspecific stimulus. These results demonstrate that males of this species responded primarily to the signal period and more or less ignored the fine temporal signal patterns. This may be adaptive when considering the potential masking of the fine syllable pattern during transmission.

Evidence for rhythm as an important signal parameter for species recognition was provided in the same species in female choice experiments. When given a choice between conspecific signals broadcast at different periods, females showed a preference for a fixed signal period of 2 s (Hartbauer et al., 2014). However, in choice tests with song models of periods < 1.5 s, females rarely approached any speaker. This is remarkable because the solo signal rate positively correlates with the energetic costs associated with song production (Hartbauer et al., 2012a). That is, if females selected males with higher signal rates they would thereby select males that invest more energy in mating displays. Their low rate of positive phonotaxis toward speakers with higher signal rates suggests stabilizing selection for the conspecific signal period.

## Female choice and the evolution of chorus synchrony

As noted above, chorus synchrony can be a by-product of species recognition if signalers in a group preserve a species-specific temporal pattern (Greenfield, 1994a). The “rhythm conservation hypothesis” is exemplified by *Neoconocephalus nebrascensis*, where the male song requires strong amplitude modulations in order to elicit a phonotactic response in females (Deily and Schul, 2009). Thus, males are forced to synchronize the amplitude modulations of their signals when in male assemblages. A similar argument for the cooperative, synchronous display of mating signals has been put forward for the synchronously-flashing firefly *Photinus carolinus*. In this case, synchrony presumably reduces the visual “clutter” caused by randomly-timed, flashing signals (Copeland and Moiseff, 2010).

Darwin (1871) noted that female preference may promote the evolution of exaggerated mating displays. The evolution of such traits could be the result of a Fisherian process in which stronger preferences and more exaggerated traits coevolve (Fisher, 1915, 1930). In most communication systems, females prefer males that advertise themselves by producing conspicuous signals that are energetically expensive to produce. This is called “Zahavi's handicap principle” after Zahavi (1975), who explained the existence of such a preference by claiming that signals are reliable indicators of male quality when their production is expensive for the signaler, and that prolonged signaling lowers the fitness of the sender (reviewed in Johnstone, 1995). The energetic costs associated with the production of acoustic signals are usually determined by at least three signal parameters: duration, amplitude, and signal rate (Prestwich, 1994; Reinhold et al., 1998; McLister, 2001; Robinson and Hall, 2002). In the context of mate choice, these signal parameters are regarded as “condition-dependent handicaps,” which indicate the quality of a sender (West-Eberhard, 1979; Andersson, 1994). Furthermore, signal traits that provide true information about the phenotypic and genetic qualities of the senders and exclude the possibility of cheating are known as “revealing handicaps” (Maynard Smith, 1985, 1991).

On the other hand, preferences for certain signal traits may be the outcome of a sensory bias in receivers that already existed before signalers evolved the traits to exploit it. In a mating context, this hypothesis suggests that, when confronted with a choice situation, females do not necessarily select males on the basis of their acoustic signal traits (indicative of male quality). Instead, certain signals can more strongly stimulate the sensory system in receivers, increasing the likelihood of mating (Ryan, 1990; Ryan et al., 1990; Kirkpatrick and Ryan, 1991; Ryan and Keddy-Hector, 1992; Arak and Enquist, 1993). For example, males of lebinthine crickets generate unusually high-frequency calls that elicit a startle response in females. In response to these calls, females generate vibratory signals that allow males to locate them (ter Hofstede et al., 2015). Arak and Enquist (1995) provided some examples in which the sensory bias in receivers creates competition between senders, with the result of more conspicuous and costly signals.

In male aggregations of anurans and katydids, females often select males on the basis of relative signal timing rather than other signal features (Greenfield, 1994b; Gerhardt and Huber, 2002). Such mating systems are especially interesting for evolutionary biologists since, by choosing males on this basis, there are no obvious direct or indirect fitness benefits for females (Alexander, 1975; Greenfield, 1994b). Any preference for a certain temporal relationship between competing signals drives the evolution of mechanisms that enable the exact timing of signals generated in a group. This “receiver bias” hypothesis suggests that synchrony or alternation has emerged as a consequence of inter-male rivalry due to inter-sexual selection (e.g., Alexander, 1975; Arak and Enquist, 1993; Greenfield, 1994a,b, 1997; Greenfield et al., 1997; Snedden and Greenfield, 1998; Gerhardt and Huber, 2002; Copeland and Moiseff, 2010). Therefore, by studying signal interactions among males in a chorus and their evaluation by receivers, one can study traits and selection at different levels. In feedback loops, traits emerge at the group level and influence the evolution of signal timing mechanisms at the individual level (Greenfield, 2015; Party et al., 2015).

### Leader preference

In male assemblages, the synchronicity of calls is usually limited in precision, with some signals leading others. Relative signal timing can enhance or reduce male attractiveness if the females exhibit a preference for a certain temporal relationship between signals displayed in imperfect synchrony. Indeed, some anurans prefer signals that are timed in advance to others (leader signals) (reviewed in Klump and Gerhardt, 1992) which was also observed in many Orthopteran species (Shelly and Greenfield, 1991; Greenfield and Roizen, 1993; Minckley and Greenfield, 1995; Galliart and Shaw, 1996; Greenfield et al., 1997; Snedden and Greenfield, 1998). Such a preference constitutes a precedence effect, which is defined as the preference for the leading signal when two closely-timed, identical signals are presented from different directions [humans (Zurek, 1987; Litovsky et al., 1999), Mammals, birds, frogs, and insects (Cranford, 1982; Wyttenbach and Hoy, 1993; Greenfield et al., 1997; Dent and Dooling, 2004; Lee et al., 2009; Marshall and Gerhardt, 2010)]. This preference may be due to the fact that the leading signal suppresses the echo (reverberation) of subsequent signals that reach the receiver in a complex acoustic environment and, thus, improves sound localization.

*Neoconocephalus spiza* is a well-studied example of a synchronizing katydid species in which females display a strong leader preference. As a consequence, individual males compete in an attempt to jam one other's signals, with synchrony emerging as an epiphenomenon (Greenfield and Roizen, 1993; Snedden and Greenfield, 1998). The observation that males regularly switch between leader and follower roles in duets, exhibiting similar “free-running” chirp periods, provides support for the hypothesis that an ongoing competition for leadership exists (Greenfield and Roizen, 1993). In this species, males stop producing unattractive follower signals within a certain critical period of time after perceiving the signals from competitors (the so-called “forbidden interval”). Unlike *N. spiza* males, males of *M. elongata* establish mostly fixed temporal relationships for their signals over long periods of time, so that individual males assume either leader or follower roles during the duet (Hartbauer et al., 2005). Even in small four-male choruses, individuals often maintain either the leader or follower role over long periods of time (Hartbauer et al., 2014). The relative timing of synchronized chirps of different males strongly influences female choice. In two-choice experiments, *M. elongata* females showed a strong preference for those chirps leading by only 70–140 ms (Fertschai et al., 2007; Hartbauer et al., 2014). There is also a trade-off between time and intensity: the advantage of a signal leading by 140 ms can be compensated by an increase in loudness of follower signals by 8 dB (for similar trade-offs in other synchronizing insects and some anuran species, see Klump and Gerhardt, 1992; Greenfield, 1994b; Howard and Palmer, 1995; Grafe, 1996; Greenfield et al., 1997; Snedden and Greenfield, 1998; Höbel, 2010). The relatively high intensity value that is necessary for leader compensation implies that females must be in close proximity to the follower to prefer this male from a chorus. As a consequence, males who persistently signal as followers in a chorus should have a reduced fitness, posing an intriguing question about the evolutionary stability of follower roles. Before discussing hypotheses that may provide an answer to this question (see Section Cooperation, Competition, and a Trade-Off between Natural and Sexual Selection), we describe an oscillator property that favors the ability of males to attain call leadership in a chorus, and results obtained from a realistic computer model of a *M. elongata* chorus.

### An oscillator property responsible for attaining leadership

Sismondo (1990) demonstrated that synchrony and alternation in *M. elongata* are consequences of song oscillator properties, which can be illustrated in the form of phase response curves. In entrainment experiments and using realistic computer models, we demonstrated that males could establish stable synchrony and bi-stable alternation of signals over a broad range of stimulus periods, covering the whole spectrum of solo chirp periods found in a male population (1.7–2.4 s; Hartbauer et al., 2005). However, the synchrony observed was not perfect, and males tended to produce their chirps as a leader only if interacting with a male that exhibited a slower intrinsic signal rate. The member of the duet with the shorter chirp period (i.e., a difference of more than 150 ms in the intrinsic signal period duration) had an increased probability of attaining leadership (Hartbauer et al., 2005). This correlation between the intrinsic signal period and lead probability has also been described in the firefly *P. cribellata* (e.g., Buck et al., 1981) and two other katydid species (Meixner and Shaw, 1986; Greenfield and Roizen, 1993).

## A realistic model of a *M. elongata* chorus

Once a realistic model of male duets had been established (Hartbauer et al., 2005), the model was extended to simulate a chorus that consisted of 15 artificial males (Hartbauer, 2008). A major advantage of this approach is that manipulations of receiver properties and chorus composition could be performed that greatly exceeded those possible in behavioral experiments. In particular, parameters such as chorus density, selective attention paid to a neighbor subset, and temporal variability of synchrony due to males joining or leaving a chorus could be modified.

The results of chorus simulations revealed that synchrony in *M. elongata* is the outcome of an ongoing phase resetting process that propels song oscillators forward and backward during every cycle. Therefore, synchrony in *M. elongata* seems to be maintained on a chirp-to-chirp basis and does not depend on the mutual adjustment of intrinsic signal periods, as in a firefly (Ermentrout, 1991) or a katydid species (Murphy et al., 2016). Even in rather complex chorus situations, in which the signal oscillators and inter-male distances between nearest neighbors varied, agents that signaled at faster intrinsic rates established the leadership position more often than other chorus members. These simulation results were confirmed in real *M. elongata* choruses that consisted of 3–4 equally spaced males. In this situation, a single male led more than 50% of all signal interactions in 68% of choruses (Hartbauer et al., 2014). A correlation could also be drawn between the intrinsic signal period and the likelihood of producing leader signals in an Indian *Mecopoda* species (Nityananda and Balakrishnan, 2007). Unlike the Malaysian *M. elongata* species, males of the Indian species also altered their intrinsic signal period to match that of their competitors, a behavior that did not allow for the establishment of consistent leader and follower roles (Nityananda and Balakrishnan, 2008).

### Manipulation of chorus density

An analysis of data from computer simulations also revealed that removing two or three agents from a synchronous chorus had only a minor effect on chorus synchrony, whereas adding agents who initially signaled at random phases greatly disturbed synchrony (Hartbauer, 2008). Therefore, in order to avoid a temporal loss of synchrony, males joining a synchronous chorus should already be phase-locked with other chorus members. Empirical evidence for such synchronous initiation of songs has recently been provided for *Neoconocephalus ensiger* (Murphy et al., 2016). Males of this katydid species seem to adjust the intrinsic signal period of their song oscillators prior to initiating the song in order to match the rate of periodic signals. Phase-locked song initiation behavior was also observed in males that were stimulated with a periodic pacer (Hartbauer, 2008). This behavior may be regarded as an adaptation to counteract the vulnerability of a synchronous chorus.

### Selective attention

Based on the results of computer simulations, Greenfield et al. (1997) argued that selective attention must be paid to a subset of males before synchrony and, especially, alternation can become a evolutionarily-stable signaling strategy. Selective attention can be gained at the neuronal, behavioral and ecological level and restricts the receivers' attention to signals broadcast by neighbors. Evidence for selective attention at the behavioral level has been provided from playback experiments conducted with alternating grasshopper and katydid species (Greenfield and Snedden, 2003). Individuals of these species need to pay selective attention to close neighbors when alternating in a chorus because, in principle, strict signal alternation is limited to only two acoustically interacting males. Evidence for selective attention at the neuronal level has been found by studying the membrane properties of individual interneurons; when signals that differ in loudness compete, the representation of the softer signal is suppressed (Pollack, 1988; Römer and Krusch, 2000). This enables receivers in a chorus to pay selective attention to the loudest signaler. Similarly, inhibitory mechanisms may result in a stronger representation of leader signals in imperfect synchrony (Nityananda et al., 2007). Despite the neuronal evidence for selective attention to leading signals, field studies indicate that the spacing of males appears to play a more important role in restricting the attention of a receiver to close neighbors (Nityananda and Balakrishnan, 2008). Simulating selective attention to only three nearest neighbors in a chorus model did not alter the likelihood of males with higher intrinsic signal rates to attain call leadership, but waves of synchronized signaling spread out among the agents (Hartbauer, 2008). This phenomenon, which is known as “wave-synchrony,” has also been observed in fireflies that flash in synchrony. It has inspired the development of a *Mecopoda*-based controller that enables the navigation of a swarm of autonomous micro-robots (Hartbauer and Römer, 2007).

## Is chorus synchrony in *M. elongata* the outcome of a sensory bias?

One proximate explanation for the preference of females for leading signals in behavior is based on a sensory bias in receivers. In the auditory system of insects, like in other vertebrates and mammals, direction-sensitive interneurons receive excitatory and inhibitory input from opposite auditory sides (review in Hedwig and Pollack, 2008). Thus, for a female receiver located between two acoustically interacting males, the signals of leader and follower males are asymmetrically represented in the auditory pathway, depending on the timed interaction of excitation and inhibition (Römer et al., 2002). Given that the leader signal has a temporal advantage, it may effectively suppress the representation of the follower signal, and the different representation of otherwise identical signals may bias the orientation of the female to the leader. The interaction of excitatory and inhibitory input may also explain quantitative values in time-intensity trading (Römer et al., 2002; Fertschai et al., 2007). In the auditory system of katydids, two interneurons that have properties favoring leading signals in a choice situation have been examined and may convey leader-biased bilateral information (Römer et al., 2002; Siegert et al., 2011). Depending on the strength of inhibition, the response to lagging signals was almost completely suppressed during the presentation of leading signals. Time-intensity-trading experiments revealed that follower signals needed a 15–20 dB advantage to compensate for the follower role, depending on the magnitude of the time difference.

However, the crucial question in the context of a possible sensory bias is whether the leader-biased response of auditory neurons evolved before or after male synchrony. It has been commonly accepted that a sensory bias can be the by-product of a sensory mechanism that evolved in a non-sexual context (Endler and McLellan, 1988; Ryan, 1990; Ryan et al., 1990; Kirkpatrick and Ryan, 1991; Ryan and Keddy-Hector, 1992; Arak and Enquist, 1993; Boughman, 2002; Arnqvist, 2006) and, therefore, that it already existed before signalers evolved traits to exploit it (“sensory exploitation” hypothesis) (Ryan and Rand, 1990, 1993; Ryan et al., 1990; Ryan, 1999). Ultimately, any bias in sensory processing with respect to closely timed signals has the potential to drive the evolution of communal signal displays toward synchrony or alternation (Greenfield, 1994a).

**Figure 3:**
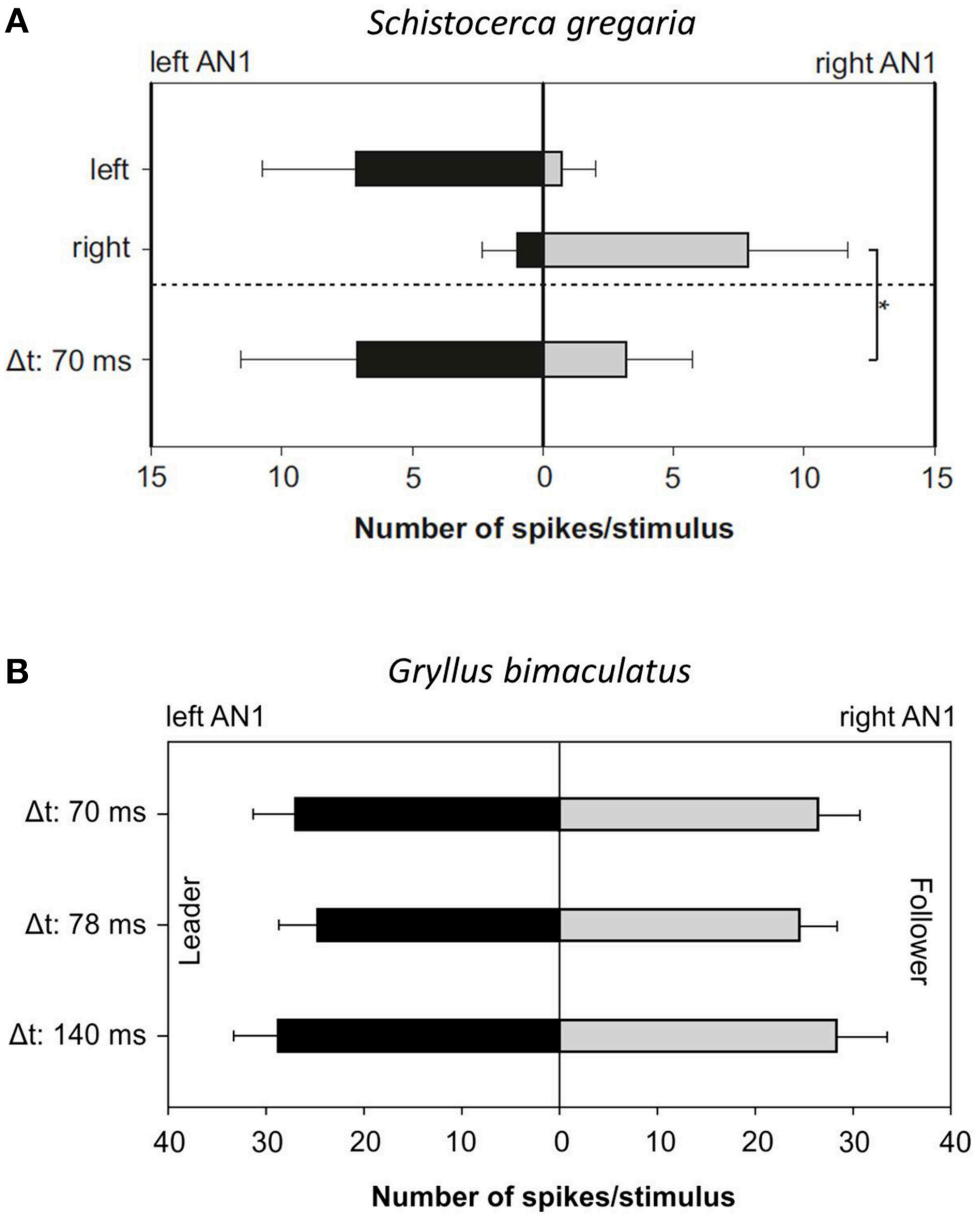
**Summary of the bilateral AN1 responses to a ***Mecopoda*** chirp in ***Schistocerca gregaria*** and ***G. bimaculatus*****. The chirp of a solo-singing *Mecopoda* was presented individually from both sides (left and right) or as a leader–follower presentation with a time lag of 70 ms. **(A)** The mean bilateral AN1 response of five *S. gregaria* individuals indicated a significantly stronger excitation on the leader side (*p* < 0.01; Mann–Whitney Rank Sum Test, Bonferroni corrected). **(B)** No significant differences at three different time delays were observed in *G. bimaculatus* (average responses obtained from 5 individuals).

Strong support for the “sensory bias” hypothesis in *Mecopoda* would be the demonstration that in distantly-related orthopteran species, where synchrony does not occur, the responses to lagging signals in directionally-sensitive interneurons are also suppressed. The results of experiments conducted with locusts and field crickets have, thus far, been ambiguous (Figure 3). A recent phylogenetic study conducted in the genus *Neconocephalus*, in which—with the exception of one species—discontinuously-calling species synchronize their calls (Greenfield, 1990; Greenfield and Schul, 2008; Deily and Schul, 2009) revealed that females do not always show a strong leader preference, which does not support the “sensory bias” hypothesis (Greenfield and Schul, 2008). The most parsimonious explanation for imperfect synchronous chorusing in *M. elongata* is that the phase change mechanism in males enables them to synchronize their chirps, and females choose leading males as a passive consequence of the precedence effect in the auditory system (see also Party et al., 2014). However, it is also possible that a feedback loop, which originated from a sensory bias, exists that gradually strengthened the leader preference once imperfect chorus synchrony had been established.

### The adaptive nature of a sensory bias

Whether a sensory bias can be adaptive or not is still a matter of debate. Female choice based on a sensory bias may provide the females with fitness benefits due to lower search costs, even if the choice does not result in offspring with superior genes that are associated with positive fitness consequences (Kirkpatrick, 1987; Guilford and Dawkins, 1991; Hill, 1994; Dawkins and Guilford, 1996). This seems to hold true for *M. elongata* females, since positive phonotaxis lasted three times longer when identical chirps were presented in strict alternation, as compared to a leader-follower situation (Fertschai et al., 2007). Such delayed responses to alternating chirps can be explained at the neuronal level, since alternating chirps elicit identical—and, thus, ambiguous—neuronal excitation on both sides, whereas leading signals cause asymmetrical responses in favor of the leader, which would allow females to reliably choose between two similar, alternative signals. Therefore, females that quickly choose from among males may enjoy fitness benefits by reducing the risk of predation that is associated with a prolonged search for mates (e.g., Belwood and Morris, 1987; Siemers and Güttinger, 2006).

The solo chirp rate of *M. elongata* is an important predictor for leadership in acoustic interactions between males. If this parameter were correlated with traits that indicated male quality such as body size or fertility, females would gain fitness benefits by choosing the leader from among a group of males. However, neither male age, body size, spermatophore volume, or the number of living offspring correlated with the solo chirp period of individual males (Hartbauer et al., 2015), corroborating the results of a nutritional study in which the solo chirp rate was shown to be a poor predictor of nutritional status (Hartbauer et al., 2006). Similarly, in the European tree frog *Hyla arborea*, the quality of males did not correlate with signal timing, although females preferentially oriented toward the first of two identical calls that overlapped in time (Richardson et al., 2008). In this frog species and in the katydid *Ephippiger ephippiger*, call leadership and overall energetic investment in acoustic signals correlated positively (Berg and Greenfield, 2005). In this respect, the systems in *H. arborea* and *E. ephippiger* are analogous to that of *M. elongata* where the probability of producing leader signals depends on a trait (intrinsic signal period) that is associated with calling energetics (Hartbauer et al., 2006), but does not correlate with indicators of male fitness. In the same way, female *E. diurnus* do not gain any obvious benefits by preferring leading calls although males are able to adjust the song oscillator phase to establish leadership (Party et al., 2014).

## Cooperation, competition, and a trade-off between natural and sexual selection

**Figure 4:**
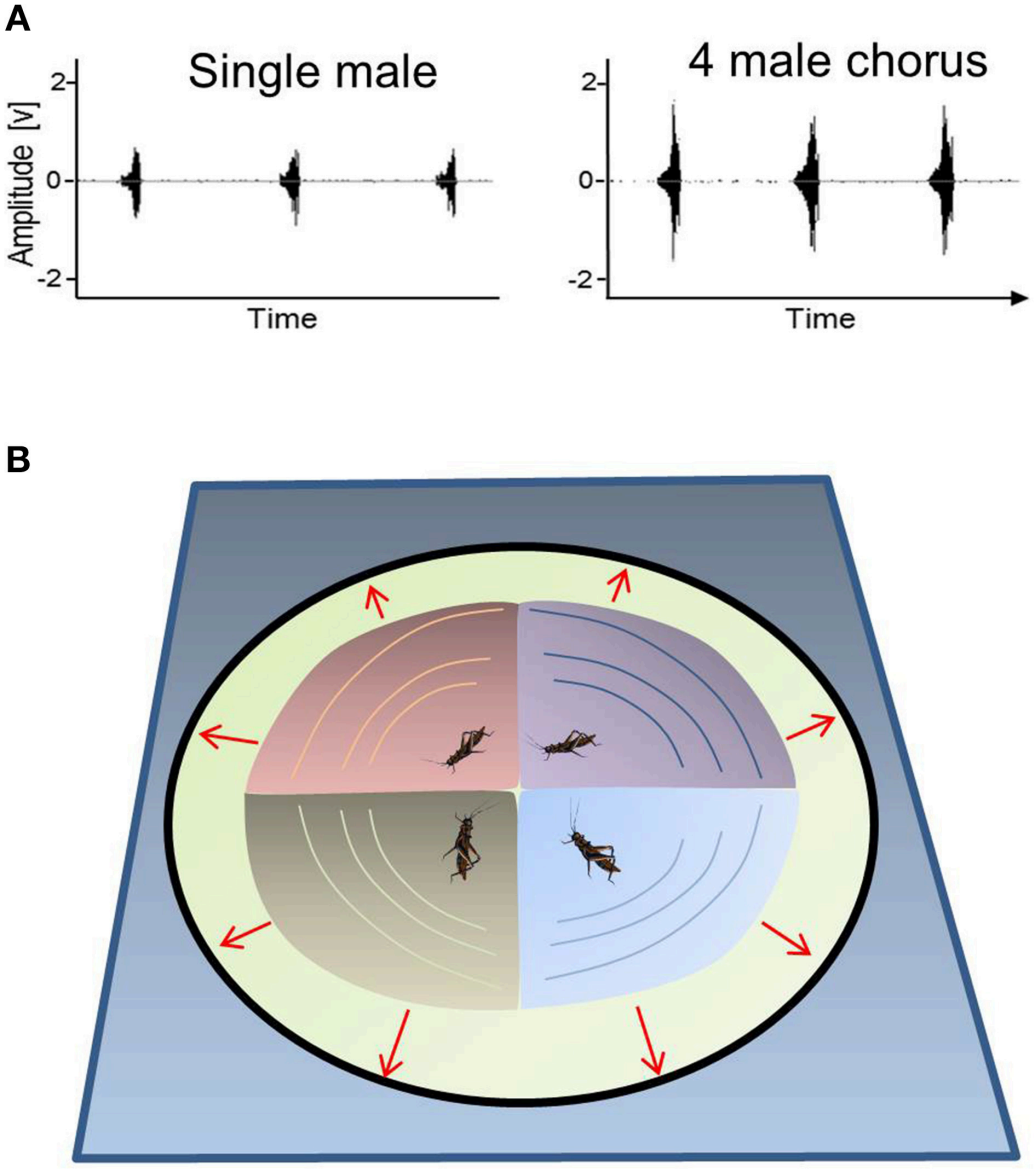
**Signal overlap in ***M. elongata*** and model of the extension of acoustic space as the result of chorus synchrony**. Four males singing in synchrony overlapped their periodic signals to a high degree. This led to a strong increase in signal amplitude **(A)** and to the enlargement of acoustic space **(B)**. In this way, a group of synchronized males can attract females from a greater distance as compared to lone singing males. In the case of signal alternation, the area in which a single male signals at higher amplitude as compared to its competitors is strongly reduced (shown as areas with different colors).

**Figure 5:**
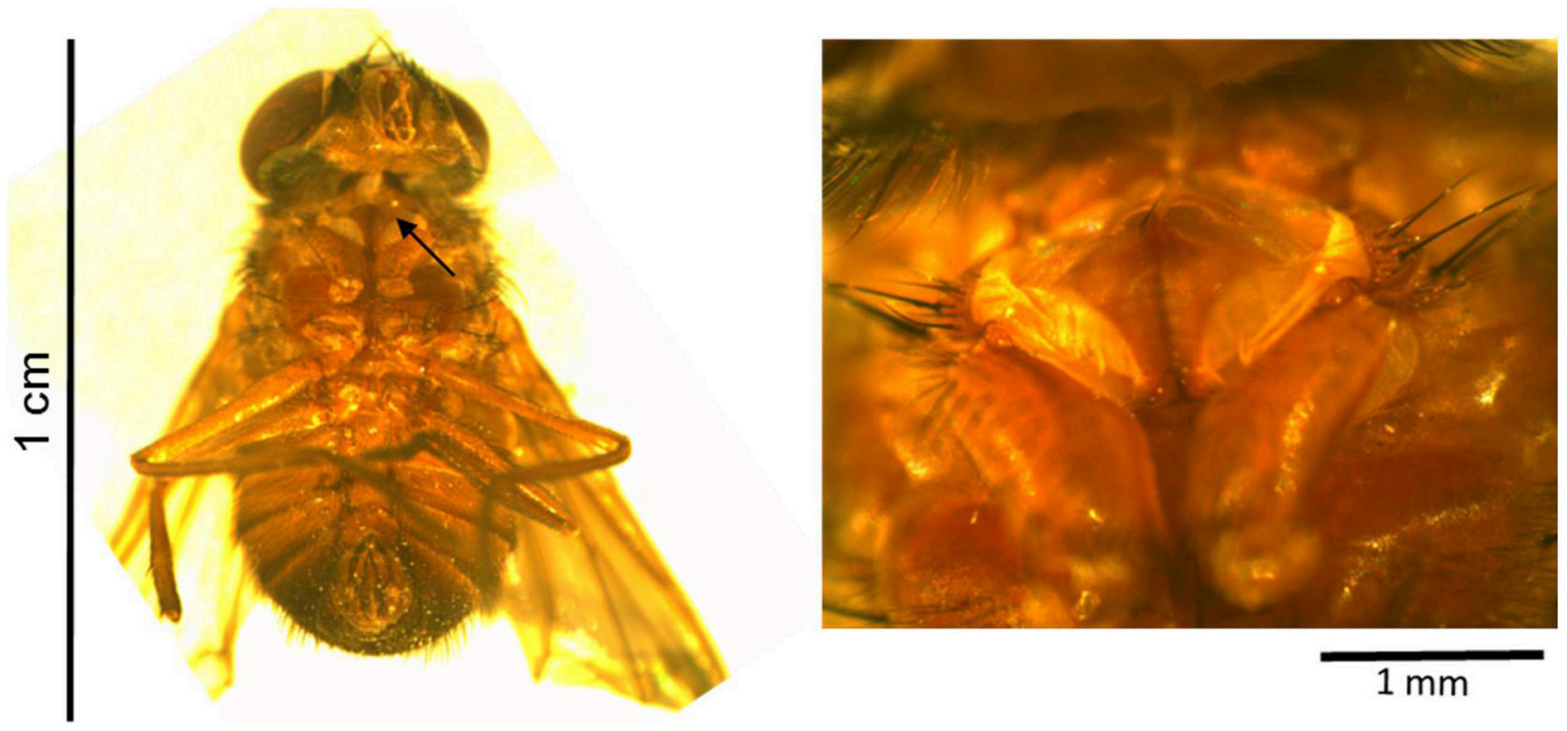
**Habitus (left) and hearing organ (right) of a female tachinid fly of an unknown ***Tachinid*** species homing in on ***M. elongata*** males**. Arrow indicates the position of the fly's ear. This fly belongs to the tribe Ormiini of an unknown genus (potentially *Therobia, Phasioormia*, or *Homotrixa*).

Why do some *M. elongata* males participate in a chorus although they are less attractive for females as followers and probably would be more successful singing in isolation? One possible explanation may be that, in some species, females prefer signals that emerge from group displays over signals produced by lone singing males, which forces males to congregate [insects (Morris et al., 1978; Cade, 1981; Doolan and Mac Nally, 1981; Shelly and Greenfield, 1991), *Hyla microcephala* (Schwartz, 1994); but see Party et al., 2015]. Choice tests performed with *M. elongata* females confirmed their preference for conspicuous group displays (Hartbauer et al., 2014). However, this result does not explain why leader and follower roles were maintained by individuals in *M. elongata* choruses, where followers were at a disadvantage due to the strong female preference for signals from leaders (Fertschai et al., 2007). Below, several alternative, although not mutually exclusive, hypotheses are presented to explain why persistent followers still exist in *M. elongata*:

1. Signaling as a follower may be beneficial when resulting from inter-male cooperation because overlapping chirps in a chorus may amplify the peak amplitude of the signals that are displayed synchronously (Figure 4A), and the resulting “beacon effect” may help distant receivers detect communal displays (see Figure 4B). In this case, females seem to evaluate the peak signal amplitude of communal displays, rather than average acoustic power. Interestingly, sound recordings revealed an elevated sound pressure level in the order of 6 dB in a chorus consisting of 3–4 acoustically-interacting *M. elongata* males (2 m nearest-neighbor distance; Hartbauer et al., 2014). Despite imperfect synchrony, the high degree of signal overlap found in this chorus situation resulted in an average increase of the root-mean-square amplitude that is almost identical to that found during the simultaneous playback of four identical, conspecific signals that perfectly overlapped in time. Given the fact that syllables comprising male chirps are interrupted by brief pauses, this result is surprising and may be attributed to signal plasticity, which is known to increase the probability of temporal overlap among the loud syllables of leader and follower signals (Hartbauer et al., 2012a). As a result, signal overlap in “four male choruses” is so high that the average duration of jointly produced signals is only 1.4 times longer (343 ms) as compared to the average signal duration of solo singing males (250 ms). It is also interesting to note that the increased signal amplitude of communal signal displays was a prerequisite for the successful simulation of the evolution of chorus synchrony in an Indian *Mecopoda* chirper, where females also preferred “leader males” (Nityananda and Balakrishnan, 2009). This observation is in contrast to results gathered for *Achroia grisella* (wax moth) leks, for which such a prerequisite does not exist (Alem et al., 2011).
  An inherent problem encountered when interpreting many group effects is the dilution of per capita mating success as compared to that of lone singing males. However, the increased amplitudes of group displays may enhance the mating probabilities of individual males if one considers the noisy background against which acoustic communication often takes place. Given these complex acoustic conditions, overlapping signals may allow individuals to increase the conspicuousness of their rhythmic signals in a group. Additionally, enhanced group signals were more attractive for females as compared to the solo song of a male (Hartbauer et al., 2014). These data suggest that chorus synchrony in *M. elongata* is the outcome of inter-male cooperation, whereby even follower males may benefit from higher mating opportunities (but see the next argument).
2. Inter-male competition for attractive leading signals may explain the high degree of signal overlap in a *Mecopoda* chorus. If chorus synchrony in *M. elongata* is the outcome of such competition, males that intrinsically produce signals more rapidly are expected to maintain similar or even slightly higher signal rates in a chorus compared to solo singing, although reduced signal rates in a chorus would facilitate signal overlap with competitors. Results obtained in small choruses consisting of 3–4 males seem to support this “competitive hypothesis” because consistent leader males increased their signal rate by 4% on average in choruses as compared to when they sang in isolation (Hartbauer et al., 2014). Therefore, the observed “beacon effect” is likely the by-product of inter-male competition for the attractive leader role rather than a cooperative effort to increase the peak signal amplitude of rhythmic communal mating displays.
3. Although inter-male competition for attractive leader signals may explain chorus synchrony, it fails to explain the evolutionary stability of followers in a *M. elongata* chorus. An alternative hypothesis suggests that sustained signaling as a follower is an evolutionary stable signaling strategy if a trade-off exists between mate attraction and conspicuousness to predators/parasitoids. In field studies, we observed a tachinid fly homing in on *M. elongata* males (Figure 5). This fly belongs to one of 13 different species of Ormiin parasitoid flies that parasitize crickets and katydids in Asia (Lehmann, 2003). Lee et al. (2009) showed that *Ormia ochracea* (Diptera, Tachinidae), a tachinid fly that parasitizes field crickets, selectively orients toward the leading of two—otherwise identical—sound sources, while the lagging source had a minimal influence on the orientation of the fly. Therefore, the parasitoid fly homing in on *M. elongata* males may exhibit a similar leader preference as *Mecopoda* females, and these males would consequently suffer higher costs when signaling as leaders (review in Zuk and Kolluru, 1998). Because parasitoids are detrimental to survival and reproduction in crickets, katydids and cicada [Crickets (Cade, 1975; Zuk et al., 1998), katydids (Lehmann and Heller, 1998) and the cicada (Lakes-Harlan et al., 2000)], this hypothesis requires further testing. Ultimately, the existence of a leader preference in parasitoid flies suggests that the maintenance of follower singing in *M. elongata* is an evolutionary stable signaling strategy that trades lower attractiveness against reduced parasitation risk. Apparently, further studies are needed to quantify the selection pressure of this parasitoid fly on the signaling system of *M. elongata*.

**Figure 6:**
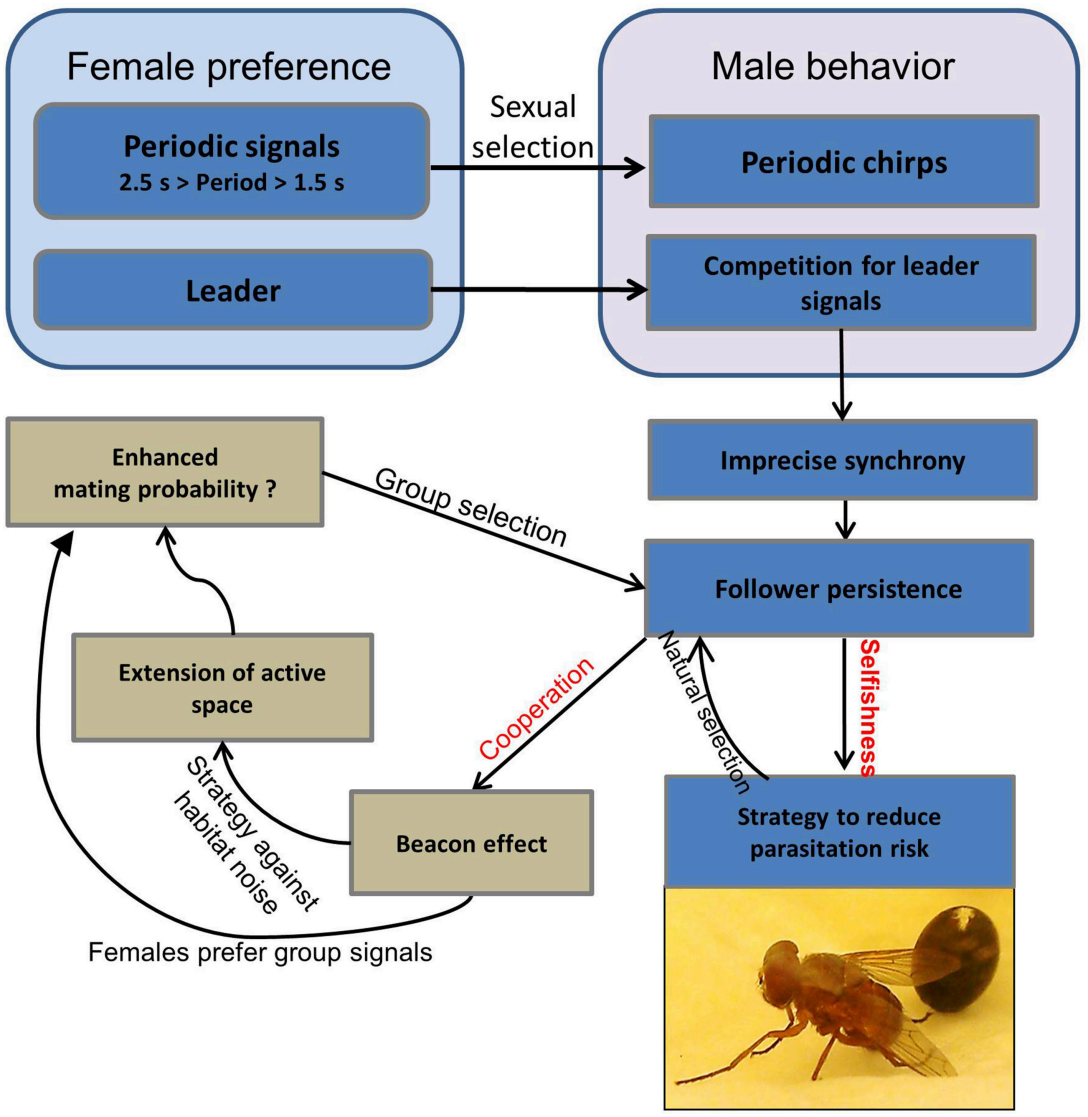
**Selection pressures potentially involved in the evolution of chorus synchrony in ***M. elongata*****. For explanation, see text (Section Cooperation, Competition, and a Trade-off between Natural and Sexual Selection). Parasitoid flies hatch from puparia (visible as the ball in the background).

A summary of various selection pressures that favor chorus synchrony in *M. elongata* is illustrated in Figure 6. Females prefer males that signal at a conspecific period of about 2 s, which forces males to synchronize their signals in a group in order to maintain this species-specific rhythm. Since females also prefer leading signals, males in a group compete for the leader role, whereby chorus synchrony emerges as a by-product (Hartbauer et al., 2014). However, chorus synchrony is imperfect and leader and follower roles often remain stable for long periods of time. The natural selection exerted by parasitoid flies that infest singing leader males may stabilize persistent follower roles. Signaling as a follower is disadvantageous in terms of reproductive success, but results in a lower risk of falling victim to a parasitoid fly (selfish strategy). Additionally, followers that persistently signal can benefit from the “beacon effect,” which extends the acoustic space in such a way as to allow females to detect conspicuous group signals. Since females more frequently approached groups producing conspicuous group signals in a choice situation as opposed to a lone singing male producing a quieter song (Hartbauer et al., 2014), males that join a synchronous chorus may increase both their mating chances and the chances of all chorus members. Additionally, computer simulations have been used to demonstrate an increase in the per capita mating possibilities for chorus members advertising themselves in a noisy acoustic environment due to strongly-operating “beacon effects” (chorus size = 4 males, inter-male distance = 10 m; Hartbauer et al., 2014). Therefore, sexual selection favors synchronous group displays, but follower roles are evolutionarily stabilized as a consequence of emergent group properties (beacon effect) and natural selection.

## Ethics statement

Insects that were used in this study were taken from a laboratory breed and do not belong to endangered species. Neurophysiological experiments have been performed in accordance with Austrian animal welfare laws.

## Author contributions

MH has drafted and written this manuscript. HR contributed with helpful comments and corrections.

## Funding

This research was funded by the Austrian Science Fund (FWF) [P21808-B09].

### Conflict of interest statement

The authors declare that the research was conducted in the absence of any commercial or financial relationships that could be construed as a potential conflict of interest.
